# Ordinal analysis applied to the results of positive matrix factorization of chemical ionization mass spectrometry data

**DOI:** 10.1016/j.mex.2020.101170

**Published:** 2020-12-04

**Authors:** Xiangrui Kong, Jan B.C. Pettersson

**Affiliations:** Department of Chemistry and Molecular Biology, Atmospheric Science, University of Gothenburg, SE-412 96 Gothenburg, Sweden

**Keywords:** Ordinal analysis, PMF, CIMS, Factor analysis, Rank map, Atmospheric science, Biomass burning, Visulization, Source appointment

## Abstract

As an innovative analytical approach ordinal analysis is applied to positive matrix factorization (PMF) analysis outputs to identify the most important species and factors in chemical ionization mass spectrometry (CIMS) data. The procedure and outcome of the ordinal analysis facilitate further automated data analysis. Prior to standard PMF analysis, CIMS data were normalized to assure equal comparisons and facilitate the analysis process. The ordinal analysis was applied to the Factor Profiles (FPs) results, where mass numbers m/z are ranked by their FP fractions. Such ranking seeks the most influential compounds leading each factor, and the top m/z can be further investigated, *e.g.* by peak assignments. Rank maps can be plotted based on the ordinal results where the FPs are converted into a different space, which can potentially be used for cluster analysis. The rank maps provide an additional method for factor identification, especially when time series or other forms of the dataset are difficult to recognize.

• Ordinal analysis identifies the most important fingerprint species leading each factor.

• Rank map visualizes the features of each factors.

• The method can be used as an online approach for source appointments of atmospheric pollutants.

## Graphical abstract

**Figure:**
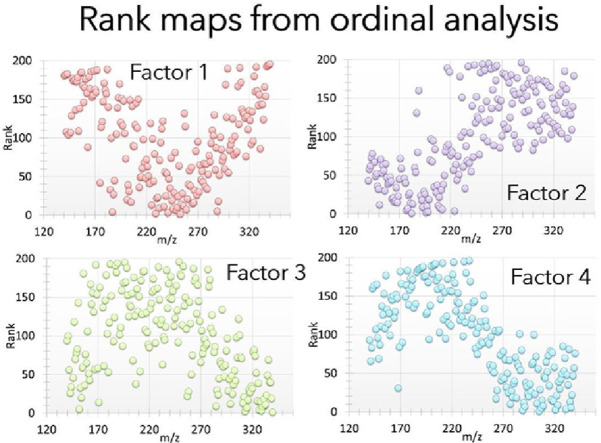

Specification table

**Table:** 

Subject area	Atmospheric Chemistry
More specific subject area	Organic aerosol; biomass burning; emission; source appointment
Method name	PMF ordinal analysis
Name and reference of original method	PMF (Paatero P, et al., 1994; 5: 111-126.)
Resource availability	The ordinal analysis can be carried out in PMF solutions from any PMF programs, and the EPA PMF 5.0 used in the study is an open source program.

## Methods

Positive Matrix Factorization (PMF) [6,7] is an analytical technique that has received widespread attention in the atmospheric science community [8], [9], [10], [11]. Here we report an innovative ordinal analysis method to be applied to standard PMF analysis outputs. The input dataset for the PMF analysis was measured by a high-resolution time-of-flight chemical ionization mass spectrometer (HR-ToF-CIMS) [3] equipped with a filter inlet for gases and aerosols (FIGAERO) [4]. The FIGAERO inlet was switched between gas and particle phase measurements with 45 min intervals. The PMF analysis was conducted with the open source program EPA PMF 5.0 [5]. The data matrix includes the peak integrals over a ± 0.5 span around each integer m/z value between m/z = 140 and m/z = 339. The CIMS data were averaged to a time resolution of 1 min.

Normalization was applied to the intensities in each nominal mass, and such process is motivated by the following aspects: (1) the normalization allows for equal comparison of all investigated signals; (2) units are eliminated, which allows for gas (time series) and particle phase (time series of thermograms) data to be analyzed by PMF simultaneously; (3) normalized data can be processed more efficiently compared to the raw data, as the latter one has huge variations in signal magnitudes. The analytical uncertainties, which dominated the overall errors, are proportional to the signal intensities [1]. Here we used a typical error, ~5% of the signal intensities, based on laboratory calibration. An example input dataset is shown in Table 1.

**Table 1:** Example of PMF input matrix.

Time	m/z 103	m/z 140	m/z 141	m/z 142	m/z 143	m/z 144
8:58	0.027	0.064	0.139	0.096	0.104	0.171
8:59	0.028	0.070	0.141	0.103	0.106	0.166
9:00	0.028	0.068	0.133	0.094	0.098	0.144
9:01	0.026	0.063	0.121	0.084	0.099	0.130
9:02	0.024	0.065	0.121	0.081	0.103	0.171
9:03	0.019	0.072	0.113	0.084	0.133	0.310
9:04	0.020	0.076	0.121	0.090	0.150	0.353
9:05	0.019	0.074	0.119	0.089	0.154	0.360
9:06	0.019	0.075	0.120	0.089	0.163	0.373
9:07	0.021	0.077	0.122	0.091	0.163	0.375

**Fig. 1:**
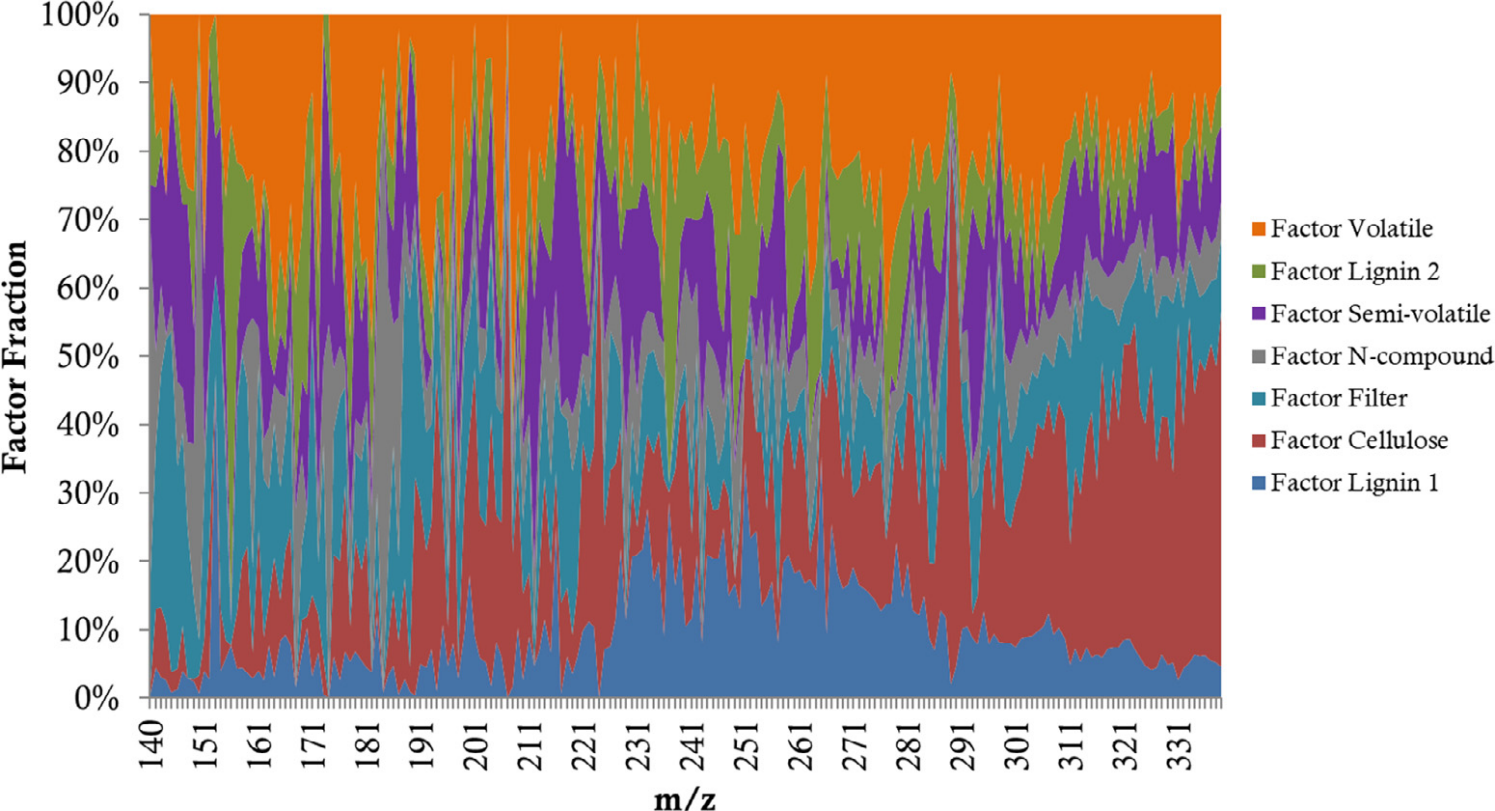
Factor profiles of a 7-factor PMF solution.

After the PMF runs, the outputs include Factor Profiles (FPs), Factor Contributions, Residuals and Run Comparisons. The ordinal analysis only involves the FP results. An example of the FP results is shown as Table 2, where the fraction of each factor in each m/z is listed in the matrix. Fig. 1 displays an example FP matrix in a color map. The CIMS data was used in the format of ± 0.5 span around each integer m/z value, which was at a low resolution. One advantage of the PMF is that the solution can indicates how much factors are influencing the nominal masses (Factor Fraction, Table 2 & Fig. 1), which related to the multiple components one would deconvolute from the high-rev MS spectra. Thus, this study shows that even without looking into the high-rev data, the PMF can reasonably reflect the composition of the low-rev mass peaks.

**Table 2:** Example of factor profile outputs (unit in %).

	Factor 1	Factor 2	Factor 3	Factor 4	Factor 5	Factor 6	Factor 7
m/z 150	7.81	10.65	3.27	3.97	25.81	25.92	22.57
m/z 151	1.69	12.09	3.81	1.67	10.91	50.24	19.60
m/z 152	35.81	8.15	3.93	0.00	3.25	40.71	8.14
m/z 153	9.49	85.80	4.57	0.14	0.00	0.00	0.00
m/z 154	25.04	10.31	5.23	0.67	0.00	43.14	15.61
m/z 155	7.37	22.56	3.92	0.88	16.67	28.52	20.07
m/z 156	3.80	55.26	0.00	0.00	28.40	10.47	2.08
m/z 157	4.53	18.52	2.39	7.84	9.53	24.29	32.90
m/z 158	8.10	13.58	1.42	6.61	4.58	28.00	37.71
m/z 159	3.15	14.09	2.55	6.99	5.08	29.71	38.43

The FP fractions directly reflect the significance of each m/z in each factor, *i.e.* how strongly they influence the factors. Therefore, for every factor, their most influential units can be identified by ranking the m/z by the fractions. An example of ranked m/z is shown in Table 3.

**Table 3:** Example of ranked m/z based on the FP values.

Rank	Factor 1		Factor 2		Factor 3		Factor 4		Factor 5		Factor 6		Factor 7	
	m/z	FP	m/z	FP	m/z	FP	m/z	FP	m/z	FP	m/z	FP	m/z	FP
**1**	190	87.61	153	85.80	173	49.52	224	74.24	103	80.36	211	59.63	207	82.39
**2**	188	51.31	251	77.42	189	39.69	200	61.30	184	56.19	146	54.22	164	58.96
**3**	201	43.25	237	67.71	217	37.76	202	53.72	174	51.19	212	52.09	194	56.45
**4**	326	39.94	265	60.27	174	37.14	322	48.21	187	31.87	151	50.24	177	55.79
**5**	152	35.81	156	55.26	229	34.05	289	47.52	168	31.80	293	48.79	208	53.22
**6**	154	25.04	263	46.98	187	31.78	226	46.30	140	31.62	148	46.77	161	52.23
**7**	191	21.05	247	41.41	219	30.54	333	45.38	178	31.03	294	43.35	181	46.58
**8**	141	19.97	249	39.21	243	30.14	321	44.84	172	30.95	154	43.14	236	46.18
**9**	186	19.30	252	38.58	144	29.68	290	44.37	182	29.75	170	42.06	204	44.14
**10**	192	17.42	170	37.91	257	29.00	336	42.62	156	28.40	198	41.37	221	43.04

The highest ranked m/z well represent the most typical species in each factor, and in-depth investigations of the high-resolution mass spectra could further identify the molecular information. Details can be found in a recent paper [2], and as an example Table 4 shows representative molecules for each factor identified by the ordinal analysis. Note that 2 of the 7 factors (Factor Lignin 1 and Factor Lignin 2, as shown in Fig. 1) were combined as 1 factor (Factor Lignin). The origins and features of the 6 factors are: Factor Cellulose (cellulose and hemicellulose pyrolysis products); Factor Volatile (end products of combustion); Factor Lignin (lignin pyrolysis products); Factor N-compounds (compounds containing nitrogen atoms); Factor Semi-volatile (compounds with intermediate volatility); Factor Filter (instrumental factor).

**Table 4:** Example of identified molecules of highest ranked m/z in various factors.

Factors	Rank	Detected Mass	Exact Mass	Formula	Possible Molecule
*Cellulose*	*1*	288.957	162.053	C6H10O5	Levoglucosan/Mannosan/Galactosan
	*2*	207.05	208.058	C7H12O7	Methyl galacturonat
	*4*	332.984	206.079	C8H14O6	Ethylidene glucose
*Volatile*	*1*	173.905	47.001	HNO2	Nitrous acid
	*2*	186.926	60.021	C2H4O2	Acetic acid
	*3*	172.91	46.006	CH2O2	Formic acid
*Lignin*	2*	250.957	124.053	C7H8O2	Guaiacol
	*3*	236.941	110.037	C6H6O2	Catechol
	*4*	264.973	138.068	C8H10O2	Creosol
*N-compounds*	*1*	103.014	104.022	C2H4N2O3	Nitroacetamide
	*2*	183.926	57.021	C2H3NO	Methyl isocyanate
	*3*	139.998	141.006	C5H3NO4	Nitrofurfural
*Semi-volatile*	*1*	154.025	155.033	C5H5N3O3	Aminomethylenebarbituric acid
	*2*	143.107	144.115	C8H16O2	Octanoic acid
	*3*	144.041	145.049	C4H7N3O3	Cytosine glycol
*Filter*	*1*	187.921	61.016	CH3NO2	Nitromethane
	*2*	188.941	62.037	C2H6O2	Ethylene glycol
	*3*	189.9	62.996	HNO3	Nitric acid

*note that the molecular identify of the 1^st^ ranked m/z cannot be assigned, thus it is not shown here. Details can be found in Kong et al. [2].

**Fig. 2:**
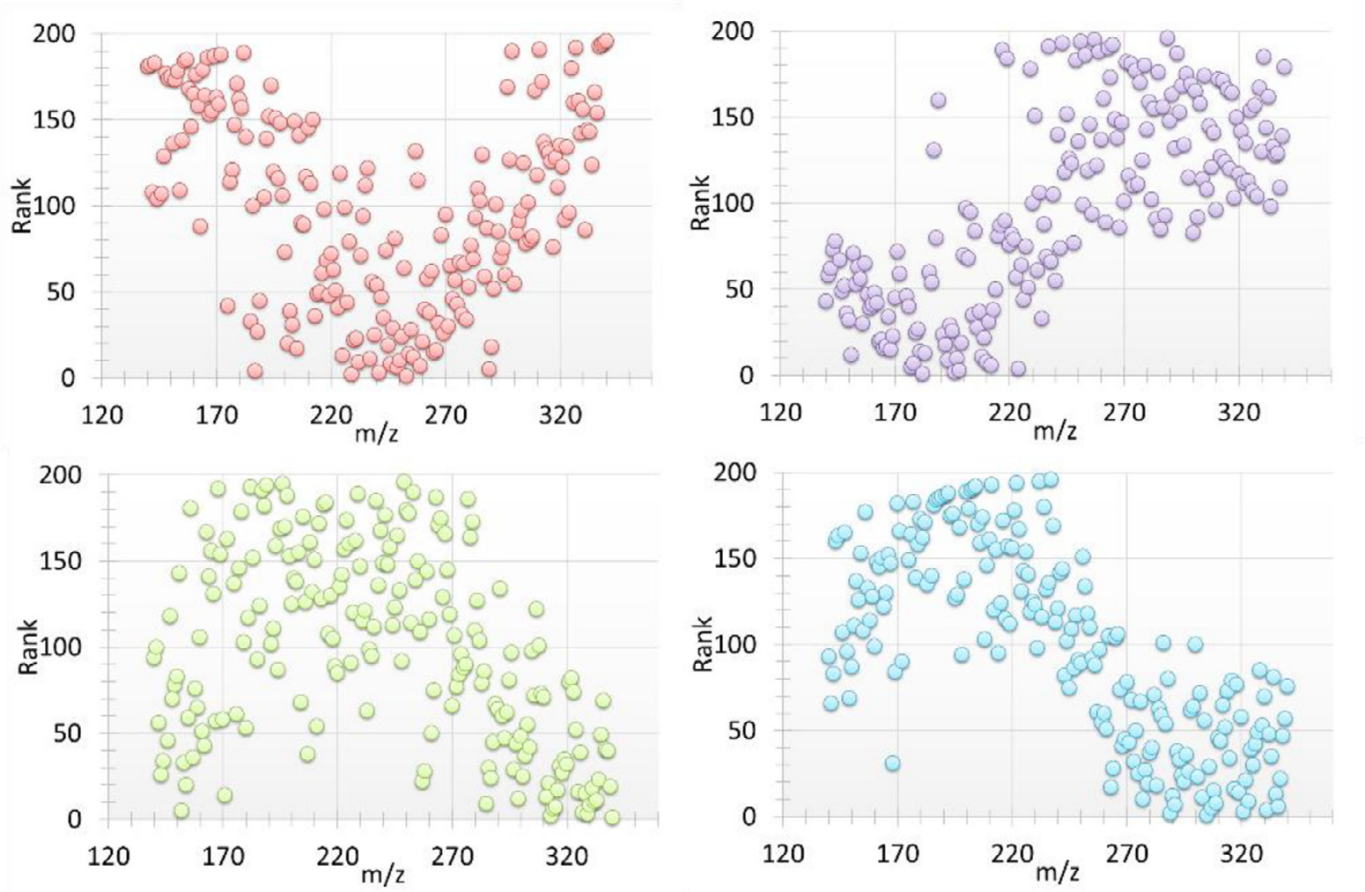
Example rank maps.

The ranking results can be visualized in rank maps. Fig. 2 shows a series of example rank maps of 4 factors, where the ranks are plotted against the m/z. Note that a high ranking results in a low position in the figures.

**Fig. 3:**
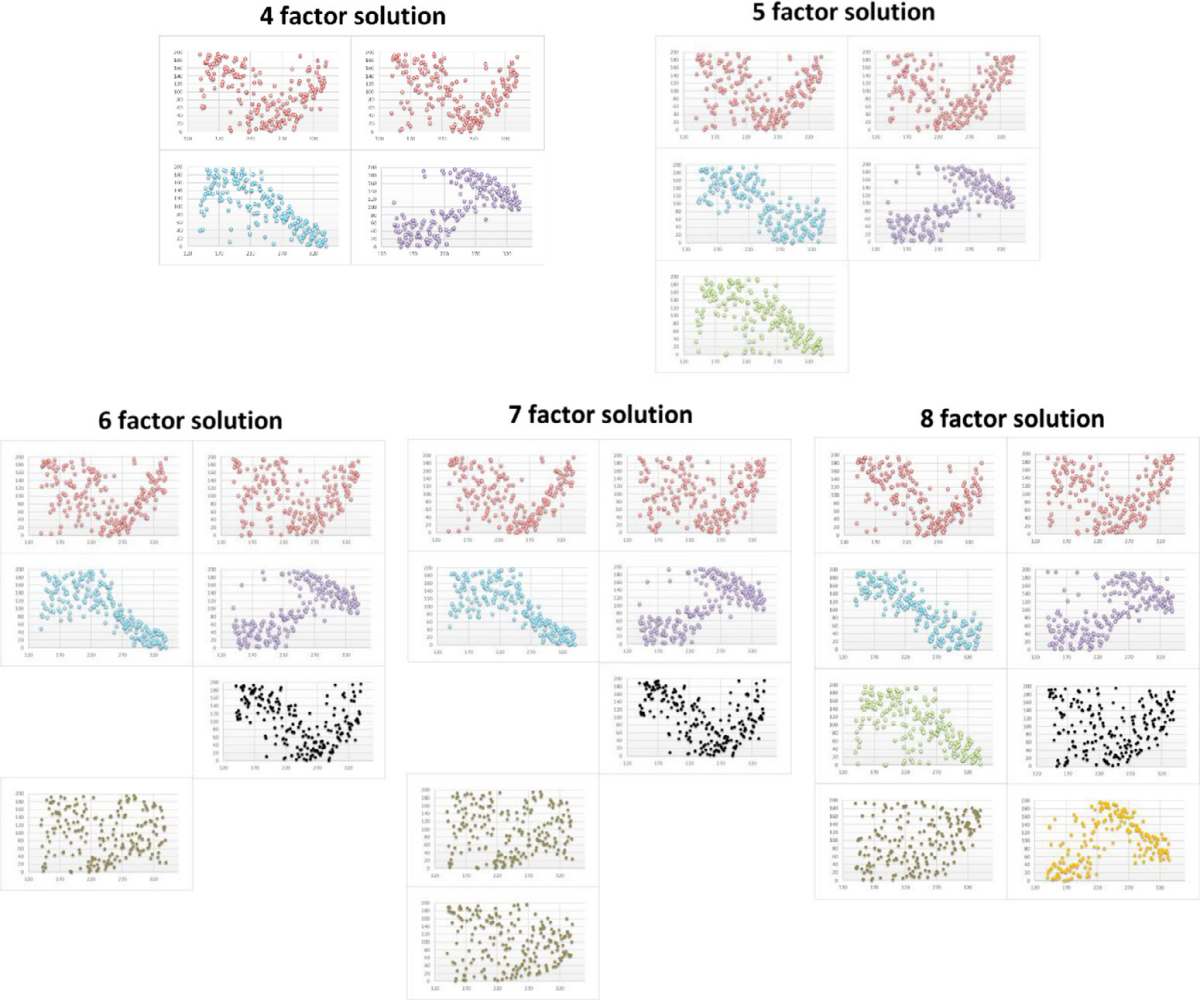
Rank maps for 4-8 factors PMF solutions.

**Fig. 4:**
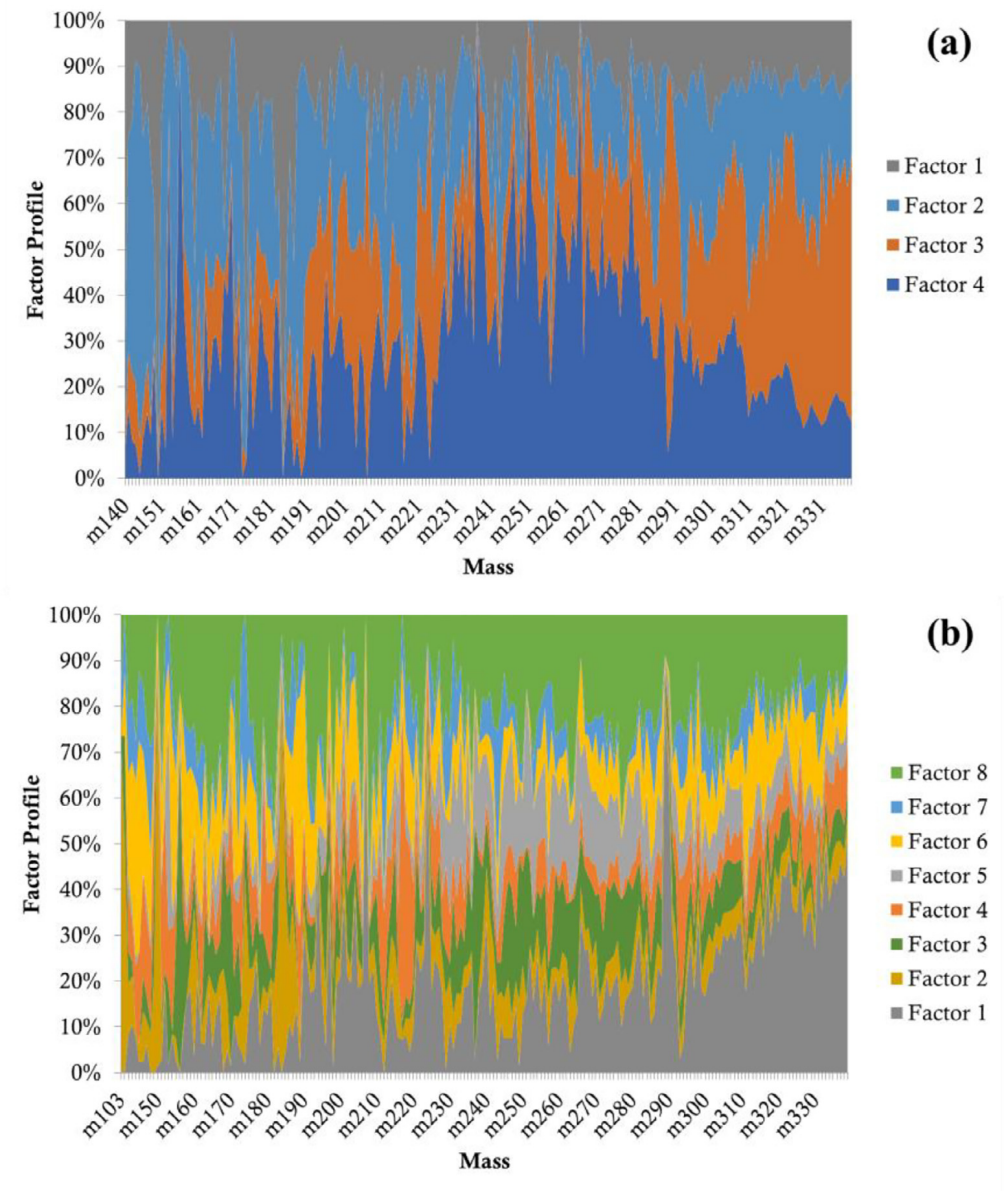
Factor profiles of 4-factor and 8-factor PMF solutions.

An important advantage of the rank maps is that the factor profiles are converted into a different space (rank versus m/z) that can be used as a 2D plot for cluster analysis. To show the advantage of the ordinal analysis, Fig. 4 shows the raw factor profiles of 4-factor and 8-factor solutions without ordinal analysis, from which it is difficult to recognize the similar factors. Moreover, in some occasions the rank maps can assist factor identification when it is difficult to recognize factors from their time series (Factor Contributions, which is one of the PMF outputs). Fig. 3 shows an example case, where some factors disappeared when increasing the number of factors (the green factor disappeared when changing from a 5-factor to a 6-factor solution), but when further increasing the number of factors the missing factor reappeared again (the green factor reappeared in the 8-factor solution). To summarize, the ordinal analysis and the rank maps provide additional ways to identify factors and fingerprint species, which has a good potential for automatic data analysis approaches related to PMF methods. One important application of this method is online analysis and source appointments of complex atmospheric pollutants, which orients quick responses during air quality monitoring.

## ORCID

Xiangrui Kong: 0000-0002-7205-0723

Jan B. C. Pettersson: 0000-0001-8420-6126

## References

[1] Allan JD, Alfarra MR, Bower KN, Williams PI, Gallagher MW, Jimenez JL (2003). Quantitative sampling using an aerodyne aerosol mass spectrometer 2. Measurements of fine particulate chemical composition in two UK cities. J. Geophys. Res..

[2] Kong X, Salvador CM, Carlsson S, Pathak R, Davidsson KO, Le Breton M (2020). Molecular characterization and optical properties of primary emissions from a residential wood burning boiler. Sci. Total Environ..

[3] Lee BH, Lopez-Hilfiker FD, Mohr C, Kurtén T, Worsnop DR, Thornton JA. (2014). An iodide-adduct high-resolution time-of-flight chemical-ionization mass spectrometer: application to atmospheric inorganic and organic compounds. Environ. Sci. Technol..

[4] Lopez-Hilfiker FD, Mohr C, Ehn M, Rubach F, Kleist E, Wildt J (2014). A novel method for online analysis of gas and particle composition: description and evaluation of a filter inlet for gases and AEROsols (FIGAERO). Atmos. Meas. Tech..

[5] Norris G, Duvall R., Brown S., Bai S. (2014). EPA Positive Matrix Factorization (PMF) 5.0 Fundamentals and User Guide.

[6] Paatero P. (1997). Least Squares formulation of robust non-negative factor analysis. Chemom. Intell. Lab. Syst..

[7] Paatero P, Tapper U. (1994). Positive matrix factorization: a non-negative factor model with optimal utilization of error estimates of data values. Environmetrics.

[8] Tao J, Zhang LM, Zhang RJ, Wu YF, Zhang ZS, Zhang XL (2016). Uncertainty assessment of source attribution of PM2.5 and its water-soluble organic carbon content using different biomass burning tracers in positive matrix factorization analysis - a case study in Beijing, China.. Sci. Total Environ..

[9] Vlachou A, Tobler A, Lamkaddam H, Canonaco F, Daellenbach KR, Jaffrezo JL (2018). Development of a versatile source apportionment analysis based on positive matrix factorization: a case study of the seasonal variation of organic aerosol sources in Estonia. Atmos. Chem. Phys. Discuss..

[10] Yan C, Nie W, Aijala M, Rissanen MP, Canagaratna MR, Massoli P (2016). Source Characterization of highly oxidized multifunctional compounds in a boreal forest environment using positive matrix factorization. Atmos. Chem. Phys..

[11] Zhang Q, Jimenez JL, Canagaratna MR, Ulbrich IM, Ng NL, Worsnop DR (2011). Understanding atmospheric organic aerosols via factor analysis of aerosol mass spectrometry: a review. Anal. Bioanal. Chem..

